# Supplementary material for: Management of Central Venous Catheters in Children and Adults on Home Parenteral Nutrition: A French Survey of Current Practice
Source: Nutrients. 2022 Jun 18;14(12):2532. doi: 10.3390/nu14122532 (PMC9227599; doi:10.3390/nu14122532)
Supplement: Supplementary file 1 [file nutrients-14-02532-s001.zip › File S1.pdf]

File S1—Survey
This is the English translation of the French original version of the online survey distributed to French HPN centers. For each question, it was possible to write a free comment.

PART I/ VENOUS ACCES CHARACTERISTICS

1. What type of central venous access do you usually use? *[Multiple Choice Question (MCQ)]*
   a. Tunneled catheter
   b. Peripherally inserted central catheter (PICC line)
   c. Totally implantable vascular device (Port-A-Cath)

2. Who performs tunneled catheter insertion? *[Free answer]. If there is more than one operator involved, indicate in which situation they are involved*

3. Do you routinely perform radiological control after CVC insertion? *[MCQ]*
   a. Yes, when placing the catheter, under image intensifier
   b. Yes, chest X-ray on return to the unit.
   c. Other

4. Do you use a clamp on the catheter? *[Single Choice Question (SCQ)]*
   a. Yes, always
   b. Yes, sometimes (specify indication)
   c. No

5. Do you use a bi-directional positive pressure valve? *[SCQ]*
   a. Yes, always
   b. Yes, sometimes (specify indication)
   c. No

6. Do you use an anti-bacterial in-line filter? *[SCQ]*
   a. Yes, always
   b. Yes, sometimes (specify indication).
   c. No

PART II/ FOLLOW-UP MODALITIES

7. Do patients (or parents in pediatrics) receive specific education regarding the detection of catheter-related complications such as obstruction and/or thrombosis? *[SCQ]*
   a. Yes, all
   b. Yes, some of them (specify indication)
   c. No

8. What is the frequency of your routine clinical check-up of patients (consultation, day hospital...)? *[Free answer]*

9. Regarding imaging monitoring,
   9.1. How often do you prescribe a venous doppler ultrasound? *[MCQ]*
      a. Never
      b. Regularly, routinely (indicate frequency)

c. When there is an incident (specify, e.g., obstruction/sepsis/other...)

9.2. How often do you prescribe a chest x-ray? *[MCQ]*

a. Never
b. Regularly, routinely (indicate frequency)
c. When there is an incident (specify, e.g., obstruction/sepsis/other...)

9.3. How often do you prescribe an angioTDM? *[MCQ]*

a. Never
b. Regularly, routinely (indicate frequency)
c. When there is an incident (specify, e.g., obstruction/sepsis/other...)

9.4. How often do you prescribe an angioMRI? *[MCQ]*

a. Never
b. Regularly, routinely (indicate frequency)
c. When there is an incident (specify, e.g., obstruction/sepsis/other...)

10. Do you change the central line systematically after a prolonged period of time, even if there are no complications?

10.1. For a tunneled catheter? *[SCQ]*

a. Yes (specify duration)
b. No
c. Not applicable (we do not use this type of venous access)

10.2. For a Port-A-Cath? *[SCQ]*

a. Yes (specify duration)
b. No
c. Not applicable (we do not use this type of venous access)

10.3. For a PICCline? *[SCQ]*

a. Yes (specify duration)
b. No
c. Not applicable (we do not use this type of venous access)

11. Regarding blood sampling,

11.1. How do you usually take blood samples? *[SCQ]*

a. On the catheter
b. Peripherally

11.2. How often do you draw blood from the catheter? *[Free answer]*

PART III/ CENTRAL VENOUS LINE OBSTRUCTION

12. Do you have a written and validated unblocking protocol? *[SCQ]*

a. Yes

b. No

13. Do you use a prophylaxis of venous access obstruction, such as locks? *[SCQ]*

a. Yes, systematically (specify the product used)
b. Yes, in some patients (specify product used AND indication)
c. No

14. Do you always prescribe imaging in cases of obstruction? *[MCQ]*

a. Yes, prior to the unblocking procedure (specify type of imaging)
b. Yes, after the unblocking procedure (specify type of imaging)
c. No

15. What molecule do you use for unblocking (Do not answer for types of venous access that you do not normally use)?

a. A tunneled catheter? *[Free answer]*
b. A PICC-line? *[Free answer]*
c. A Port-A-Cath? *[Free answer]*

16. What do you suggest in case of failure of the unblocking procedure?

a. Repeat the procedure (if yes, specify up to how many times)
b. Change of catheter
c. Other (specify)

PART IV/ THROMBO-EMBOLIC RISK

17. In which situation do you test for thrombophilia? *[MCQ]*

a. Before HPN onset, always.
b. Before HPN onset, in certain patients (specify indication)
c. After diagnosis of CRT, always
d. After diagnosis of CRT, in certain patients (specify indication)
e. Never

18. In which of the following situations would you prescribe venous imaging? *[MCQ]*

a. Systematically before a first CVC (specify the type of imaging)
b. After a thromboembolic event (specify the type of imaging)
c. In case of catheter related blood stream infection (specify type of imaging)
d. In case of unexplained biological inflammation, (specify type of imaging)
e. Before CVC replacement (specify type of imaging)

19. Do certain patients receive prophylactic anticoagulation? *[SCQ]*

a. Yes
b. No

19.1. If so, please specify

a. For which indication? (For example: All patients, thrombophilia, history of thrombosis, use of risk score ...) *[Free answer]*
b. Which molecule? *[Free answer]*
c. According to which scheme/modality? *[Free answer]*

20. Do you have any indications for long-term full-dose anticoagulation? *[MCQ]*
a. No
b. Yes, persistent thrombosis after treatment
c. Yes, abnormal thrombophilia test
d. Yes, other (specify)

PART V/ MANAGEMENT OF VENOUS THROMBOSIS

21. Regarding the prescription of anticoagulation drugs, what is your usual approach? *[MCQ]*
a. Use of a written and validated internal protocol
b. Systematically ask for a specialist advice (please specify the specialty)
c. Occasional request for specialist advice (please specify the specialty)

22. Regarding the management of a recent acute deep vein thrombosis
a. Which molecule do you typically use as first-line therapy? *[Free answer]*
b. What is the usual duration of anticoagulation? *[Free answer]*
c. Do you routinely control imaging, and if so, when? *[Free answer]*

23. How do you manage an incidentally detected asymptomatic CRT? *[SCQ]*
a. Full-dose anticoagulation (specify duration of treatment and drug).
b. Prophylactic anticoagulation (specify duration of treatment and drug).
c. Withholding of treatment
d. Request for specialist advice

24. Do you use pharmacological monitoring to adjust the anticoagulant dose? *[MCQ]*
a. Yes, systematically for VKAs (INR)
b. Yes, systematically for LMWH (Anti-Xa)
c. Yes, in certain cases (specify, e.g.: ineffectiveness of treatment, bleeding, etc.)

4.1. If so, would you say that the management of anticoagulants is difficult? *[SCQ]*
a. Yes
b. No
