# Supplementary material for: Management of Central Venous Catheters in Children and Adults on Home Parenteral Nutrition: A French Survey of Current Practice
Source: Nutrients. 2022 Jun 18;14(12):2532. doi: 10.3390/nu14122532 (PMC9227599; doi:10.3390/nu14122532)
Supplement: Supplementary file 1 [file nutrients-14-02532-s001.zip › Table S1.pdf]

Table S1. Indications of venous vascular imaging

Clinical situation	Pediatric centers (n=21)	Adult centers (n=13)	*p*
Before a first CVC	3 (14%)	3 (23%)	0.85
d-US	3	3	
Thrombosis	15 (71%)	9 (69%)	1
d-US	12	7	
Angio CT	2	4	
Angio MR	2	0	
CRBSI	6 (29%)	5 (38%)	0.82
d-US	5	5	
TTE	2	0	
Unexplained biological inflammation	4 (19%)	3 (23%)	1
d-US	4	1	
TTE	1	0	
PET	0	1	
Angio CT	0	2	
Before CVC replacement	16 (76%)	6 (46%)	0.16
d-US	15	5	
Angio CT	1	3	

CVC: central venous catheter. d-US: Doppler ultrasound. CT: computed tomography. MR: magnetic resonance. CRBSI: catheter related blood stream infection. TTE: trans thoracic echocardiography. PET: positron emission tomography.
